# Association of insulin resistance, from mid-life to late-life, with aortic stiffness in late-life: the Atherosclerosis Risk in Communities Study

**DOI:** 10.1186/s12933-020-0986-y

**Published:** 2020-01-28

**Authors:** Anna K. Poon, Michelle L. Meyer, Hirofumi Tanaka, Elizabeth Selvin, James Pankow, Donglin Zeng, Laura Loehr, Joshua W. Knowles, Wayne Rosamond, Gerardo Heiss

**Affiliations:** 1 Department of Epidemiology, University of North Carolina Gillings School of Global Public Health, Chapel Hill, USA; 2 Department of Emergency Medicine, University of North Carolina School of Medicine, Chapel Hill, USA; 3 Department of Kinesiology and Health Education, University of Texas at Austin, Austin, USA; 4 Department of Epidemiology, Johns Hopkins Bloomberg School of Public Health, Baltimore, USA; 5 Division of Epidemiology and Community Health, University of Minnesota, Minneapolis, USA; 6 Department of Biostatistics, University of North Carolina Gillings School of Global Public Health, Chapel Hill, USA; 7 Department of Medicine and Cardiovascular Institute, Stanford University, Stanford, USA; 8 1620 Tremont Street, OBC 3-34, Boston, MA 02120 USA

**Keywords:** Insulin resistance, Homeostatic model assessment of insulin resistance, Triglyceride to high-density lipoprotein cholesterol ratio, Triglyceride and glucose index, Arterial stiffness, Aortic stiffness, Carotid-femoral pulse wave velocity

## Abstract

**Background:**

Insulin resistance may contribute to aortic stiffening that leads to end-organ damage. We examined the cross-sectional association and prospective association of insulin resistance and aortic stiffness in older adults without diabetes.

**Methods:**

We analyzed 2571 men and women at Visit 5 (in 2011–2013), and 2350 men and women at repeat examinations from baseline at Visit 1 (in 1987–1989) to Visit 5 (in 2011–2013). Linear regression was used to estimate the difference in aortic stiffness per standard unit of HOMA-IR, TG/HDL-C, and TyG at Visit 5. Linear mixed effects were used to assess if high, as opposed to non-high, aortic stiffness (> 75th percentile) was preceded by a faster annual rate of change in log-HOMA-IR, log-TG/HDL-C, and log-TyG from Visit 1 to Visit 5.

**Results:**

The mean age of participants was 75 years, 37% (n = 957) were men, and 17% (n = 433) were African American. At Visit 5, higher HOMA-IR, higher TG/HDL-C, and higher TyG were associated with higher aortic stiffness (16 cm/s per SD (95% CI 6, 27), 29 cm/s per SD (95% CI 18, 40), and 32 cm/s per SD (95% CI 22, 42), respectively). From Visit 1 to Visit 5, high aortic stiffness, compared to non-high aortic stiffness, was not preceded by a faster annual rate of change in log-HOMA-IR from baseline to 9 years (0.030 (95% CI 0.024, 0.035) vs. 0.025 (95% CI 0.021, 0.028); p = 0.15) or 9 years onward (0.011 (95% CI 0.007, 0.015) vs. 0.011 (95% CI 0.009, 0.013); p = 0.31); in log-TG/HDL-C from baseline to 9 years (0.019 (95% CI 0.015, 0.024) vs. 0.024 (95% CI 0.022, 0.026); p = 0.06) or 9 years onward (− 0.007 (95% CI − 0.010, − 0.005) vs. − 0.009 (95% CI − 0.010, − 0.007); p = 0.08); or in log-TyG from baseline to 9 years (0.002 (95% CI 0.002, 0.003) vs. 0.003 (95% CI 0.003, 0.003); p = 0.03) or 9 years onward (0 (95% CI 0, 0) vs. 0 (95% CI 0, 0); p = 0.08).

**Conclusions:**

Among older adults without diabetes, insulin resistance was associated with aortic stiffness, but the putative role of insulin resistance in aortic stiffness over the life course requires further study.

## Background

Insulin resistance is defined by a reduced response to insulin in tissues [1] that is associated with adverse health risks, including diabetes [2], coronary heart disease [3], reduced cognitive function [4], and reduced renal function [5]. Because insulin resistance is an antecedent to metabolic dysregulation and metabolic disorders that contribute to adverse health risks, insulin resistance represents an important target for primary prevention.

Aortic stiffness can damage end organs such as the heart, the brain, and the kidneys [6]. Studies suggest that central arterial stiffness is greater in adults with diabetes [7, 8] and, among adults without diabetes, central arterial stiffness is greater with greater insulin resistance [8–12]. This evidence suggests insulin resistance, a precursor to most diabetes, may contribute to central arterial stiffening. It remains to be determined whether the natural course of insulin resistance since mid-life is associated with central arterial stiffness in older adults.

Insulin resistance can be estimated with insulin resistance indexes that are less invasive than standard reference methods [13]. The homeostatic model assessment of insulin resistance (HOMA-IR) is often used in research and correlated with direct measures of insulin mediated glucose uptake [14], but its application is hampered by lack of standardization of insulin assays [15]. The triglyceride to high-density lipoprotein cholesterol ratio (TG/HDL-C) and triglyceride and glucose index (TyG) are similarly correlated with direct measures [16, 17] and predictive of diabetes [18], but in contrast benefit from the well established standardization of lipid assays. A comparison of HOMA-IR, TG/HDL-C, and TyG as indexes of insulin resistance in exposure-outcome associations may inform their use in future studies.

Our goals were to: (1) assess the cross-sectional association of HOMA-IR, TG/HDL-C, and TyG with aortic stiffness in late-life; (2) assess the prospective association of HOMA-IR, TG/HDL-C, and TyG, since mid-life, with aortic stiffness in late-life; and (3) compare HOMA-IR, TG/HDL-C, and TyG in their cross-sectional and prospective associations with aortic stiffness, in older adults without diabetes.

## Methods

### Study population

The Atherosclerosis Risk in Communities Study is an ongoing prospective cohort of 15,792 participants ages 45 to 64, recruited in 1987–1989 from four US communities: Washington County, Maryland; Forsyth County, North Carolina; Jackson, Mississippi; and the northwestern suburbs of Minneapolis, Minnesota [19]. Cohort exams were conducted at Visit 1 (1987–1989), Visit 2 (1990–1992), Visit 3 (1993–1995), Visit 4 (1996–1998), Visit 5 (2011–2013), and Visit 6 (2016–2017); other exams are ongoing. Prior to exams, participants were asked to fast for ≥ 8 h, to refrain from smoking and vigorous exercise, and to bring medications used in the prior 2 weeks. The study was approved by the institutional review boards at all field centers of the ARIC Study and informed consent was obtained from all participants.

Aortic stiffness was measured on 5683 participants out of 6538 participants attending Visit 5. For the cross-sectional analysis using Visit 5, we excluded participants with: (1) missing carotid-femoral pulse wave velocity (n = 555); (2) diabetes or missing diabetes status (n = 1426 and n = 673, respectively); (3) missing values for either insulin resistance index (n = 254); (4) body mass index ≥ 40 kg/m^2^ (n = 47); (5) major arrhythmia defined by Minnesota code 8-1-3, 8-3-1, or 8-3-2 (n = 81); (6) aortic revascularization (n = 28); (7) biased waveforms identified by expert review and Minnesota code 8-1-2 (n = 7); 8) aortic stenosis (n = 13); (9) aortic regurgitation (n = 15); and (10) carotid-femoral pulse wave velocity ± 3 standard deviations from the mean (n = 13). For the prospective analysis using Visits 1, 4, and 5, we further excluded participants missing either insulin resistance index at Visit 1 and Visit 4 (n = 0 and n = 221, respectively) based on exclusions described in Additional file 1: Method S1. The corresponding analytic samples included 2571 participants for the cross-sectional analysis and 2350 participants for the prospective analysis.

### Blood collection, processing, and assays

Blood specimens were collected at Visits 1, 4, and 5, using a standardized venipuncture protocol, processed within 90 min, and shipped weekly to central laboratories. Fasting glucose was assayed using enzymatic methods. Fasting insulin was assayed using immunoassay methods. Triglyceride was assayed using enzymatic methods. High-density lipoprotein cholesterol was assayed using precipitation methods and direct methods. Split samples were analyzed for quality control. At Visit 5, the coefficient of variation was 3.1% (mean 112.9 mg/dL) for fasting glucose; 10.6% (mean 12.9 μU/mL) for fasting insulin; 4.9% (mean 125.2 mg/dL) for triglyceride; and 4.2% (mean 51.7 mg/dL) for high-density lipoprotein cholesterol. Assays are described in detail in Additional file 1: Table S1. To address bias due to assay drift, high-density lipoprotein cholesterol was re-calibrated [20].

### Insulin resistance indexes

The homeostatic model assessment of insulin resistance (HOMA-IR) was estimated at Visits 1, 4, and 5, as (fasting glucose in mg/dL) multiplied by (fasting insulin in μU/mL) divided by 405. The triglyceride to high-density lipoprotein cholesterol ratio (TG/HDL-C) was calculated at Visits 1, 4, and 5, as: (triglyceride in mg/dL) divided by (high-density lipoprotein cholesterol in mg/dL). The triglyceride and glucose index (TyG) was calculated at Visits 1, 4, and 5, as: Ln [(fasting triglyceride in mg/dL × fasting glucose in mg/dL)/2]. The standard deviation was 1.6 for HOMA-IR, 1.3 for TG/HDL-C, and 0.4 for TyG. In a short-term repeatability study conducted $\approx$ 4–8 weeks apart at Visit 5, the intraclass correlation coefficient was 0.70 for HOMA-IR and 0.80 for TG/HDL-C [21].

### Pulse wave velocity

Carotid-femoral pulse wave velocity was measured at Visit 5, in the supine position using the VP-1000 Plus (Omron, Kyoto, Japan) device [22]. Pulse waveforms were acquired in the common carotid and common femoral artery for 30 s by applanation sensors. Pulse wave travel distance was equal to: (the distance from the carotid artery to the femoral artery in cm) minus (the distance from the carotid artery to the suprasternal notch in cm). Time was equal to the time delay between the foot of the proximal and distal waveforms; time was automatically detected by the device. Pulse wave velocity was equal to: (the distance in cm) divided by (the time in s).

### Covariates

Standardized procedures and interviews were implemented by trained staff and technicians at each examination visit [22, 23]. Waist circumference was measured in centimeters. Body mass index was equal to: (weight in kg) divided by (standing height in m)^2^. Blood pressure was measured in a seated position using a sphygmomanometer; the mean was calculated for the last two of three measurements. Mean arterial pressure was equal to: (1/3)(systolic blood pressure in mmHg) + (2/3)(diastolic blood pressure in mmHg). Heart rate was measured in beats per minute. Self-report was used to determine current smoker status (yes vs. no), current drinker status (yes vs. no), former smoker status (yes vs. no), and former drinker status (yes vs. no). Diabetes was defined by fasting glucose ≥ 126 mg/dL, non-fasting glucose ≥ 200 mg/dL, use of diabetes medication, or self-reported physician diagnosis of diabetes.

### Statistical analysis

Participant characteristics were described by quartiles of HOMA-IR, TG/HDL-C, and TyG at Visit 5. For the cross-sectional analysis at Visit 5, linear regression was used to estimate the difference and 95% confidence interval (difference, 95% CI) in aortic stiffness per standard unit of the index. Logistic regression was used to estimate the odds ratio and 95% confidence interval (OR, 95% CI) for high aortic stiffness (> 75th percentile) per standard unit of the index. A test for interaction was used to assess heterogeneity by gender (equal to the product term between gender and the standardized index) and a p-value < 0.10 was considered nominally statistically significant.

For the prospective analysis from Visits 1, 4, to 5, linear mixed effects were used to estimate the annual rate of change and 95% CI in log-transformed index. There was non-linearity in the annual rate of change in log-TG/HDL-C, so a linear spline term at 9 years was included for log-HOMA-IR, log-TG/HDL-C, and log-TyG. A test for interaction was used to assess heterogeneity by high aortic stiffness (equal to the product term between high aortic stiffness and time) and a p-value < 0.10 was considered nominally statistically significant. For ease of interpretation, the change per year was re-expressed as the percent change per year, equal to: ($e^{\beta }$ − 1) × 100.

We addressed potential bias due to informative censoring from diabetes and death with the use of shared parameter models as set out in Additional file 1: Method S2. For log-HOMA-IR, the annual rate of change increased from 0.019 to 0.023 from baseline to 9 years as a result of addressing potential bias; after 9 years, there was an increase from 0.010 to 0.014. For log-TG/HDL-C, the annual rate of change increased from 0.017 to 0.018 from baseline to 9 years as a result of addressing potential bias; after 9 years, there was an increase from − 0.008 to − 0.007. Addressing potential bias identified steeper annual rates of change. However, on average, it was minimal and deemed ignorable.

We examined the cross-sectional associations and prospective associations by percent change in abdominal adiposity. Percent change in abdominal adiposity was equal to the percent change in waist circumference, with percent change ≥ 0% defined as gain and percent change < 0% defined as loss.

All analyses were adjusted for age, gender (except for gender-specific estimates), and race/study-site. Analyses were conducted in SAS version 9.4. Additional analyses using shared parameter models were conducted in R version 3.5.1 [24].

## Results

The analytic sample included participants ages 67 to 90 years, without diabetes, at Visit 5. At Visit 5, the mean age of participants was 75 years, 37% (n = 957) were men, and 17% (n = 433) were African American. Waist circumference, body mass index, and diastolic blood pressure were higher with higher quartiles of HOMA-IR (Table 1); similar trends were seen with waist circumference and body mass index, but not diastolic blood pressure by quartiles of TG/HDL-C and TyG (Additional file 1: Tables S2 and S3). The mean follow-up time was 24 years (range: 21 to 26 years).

**Table 1:** Characteristics of men and women ages 67–90 (n = 2571) by quartiles of HOMA-R (Visit 5, 2011–2013)

	HOMA-IR				Overall Mean ± SE or n (%)
	Quartile 1 Mean ± SE or n (%)	Quartile 2 Mean ± SE or n (%)	Quartile 3 Mean ± SE or n (%)	Quartile 4 Mean ± SE or n (%)	
Demographic					
Age, years	75 ± 0.2	75 ± 0.2	76 ± 0.2	75 ± 0.2	75 ± 0.1
Men	195 (30)	235 (37)	250 (39)	277 (43)	957 (37)
African American	88 (14)	109 (17)	102 (16)	134 (21)	433 (17)
Anthropometric					
Waist circumference, cm	90 ± 0.4	95 ± 0.4	99 ± 0.4	103 ± 0.4	97 ± 0.2
BMI, kg/m^2^	24 ± 0.1	26 ± 0.1	28 ± 0.2	30 ± 0.2	27 ± 0.1
Hemodynamic					
SBP, mmHg	130 ± 0.7	130 ± 0.7	131 ± 0.7	130 ± 0.6	130 ± 0.3
DBP, mmHg	65 ± 0.4	67 ± 0.4	66 ± 0.4	68 ± 0.4	66 ± 0.2
MAP, mmHg	87 ± 0.5	88 ± 0.4	88 ± 0.4	88 ± 0.4	88 ± 0.2
Heart rate, beats per minute	62 ± 0.4	64 ± 0.4	64 ± 0.4	65 ± 0.4	64 ± 0.2
Blood pressure medication	333 (52)	378 (59)	449 (70)	480 (75)	1640 (64)
Behavioral					
Current smoker	46 (7)	33 (5)	39 (6)	23 (4)	141 (6)
Current drinker	397 (63)	368 (59)	357 (56)	305 (48)	1427 (56)
Former smoker	279 (46)	295 (50)	289 (48)	312 (53)	1175 (49)
Former drinker	127 (20)	131 (21)	162 (25)	195 (31)	615 (24)
cfPWV, cm/s					
Men	1096 ± 21	1173 ± 19	1161 ± 18	1135 ± 17	1143 ± 9
Women	1089 ± 14	1103 ± 14	1117 ± 14	1147 ± 15	1112 ± 7
Overall	1091 ± 12	1129 ± 12	1134 ± 11	1142 ± 11	1124 ± 6
High cfPWV, > 75th percentile					
Men	44 (23)	76 (32)	68 (27)	65 (23)	253 (26)
Women	83 (19)	95 (23)	111 (28)	98 (27)	387 (24)
Overall	127 (20)	171 (27)	179 (28)	163 (25)	640 (25)

Characteristics are defined by either the mean ± standard error or the frequency (percent). For HOMA-IR quartiles, the lower and upper limits are: [0.04, 1.58) for quartile 1; [1.58, 2.41) for quartile 2; [2.41, 3.60) for quartile 3; and [3.60, 9.23] for quartile 4

*BMI* body mass index, *SBP* systolic blood pressure, *DBP* diastolic blood pressure, *MAP* mean arterial pressure, *cfPWV* carotid-femoral pulse wave velocity, *HOMA-IR* homeostatic model assessment of insulin resistance

**Fig. 1:**
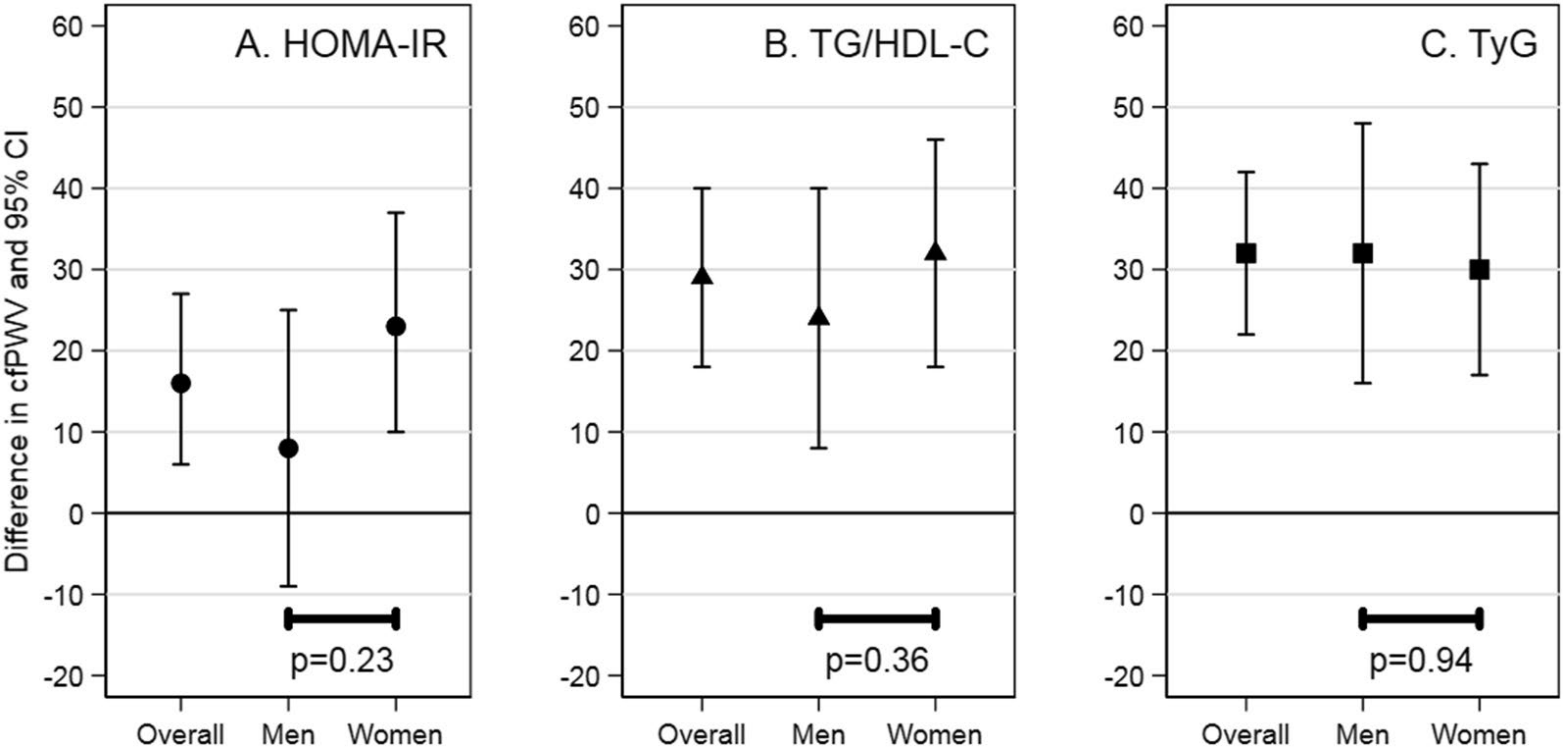
Cross-sectional association of insulin resistance indexes with aortic stiffness in adults ages 67–90. *HOMA*-*IR* homeostatic model assessment of insulin resistance, *TG/HDL*-*C* triglyceride to high-density lipoprotein cholesterol ratio, *TyG* triglyceride and glucose index, *cfPWV* carotid-femoral pulse wave velocity. The difference and 95% CI are interpreted as the difference in aortic stiffness per standard deviation increment in insulin resistance index. The test for interaction is the p-value for the product term of insulin resistance index, that has been standardized, and gender. Estimates are adjusted for age, gender (except for gender-specific estimates), and race/study site. The standard deviation was 1.6 for HOMA-IR, 1.3 for TG/HDL-C, and 0.4 for TyG

In the cross-sectional analysis, higher HOMA-IR, higher TG/HDL-C, and higher TyG were associated with higher aortic stiffness. Specifically, mean aortic stiffness was higher per standard unit of HOMA-IR (difference: 16 cm/s per SD (95% CI 6, 27)), TG/HDL-C (difference: 29 cm/s per SD (95% CI 18, 40)), and TyG (difference: 32 cm/s per SD (95% CI 22, 42)). The estimates were higher for women than men for HOMA-IR, higher for women than men for TG/HDL-C, and similar for women and men for TyG, but the differences were not statistically significant (p = 0.23, p = 0.36, and p = 0.94, respectively) (Fig. 1; Additional file 1: Table S4).

**Fig. 2:**
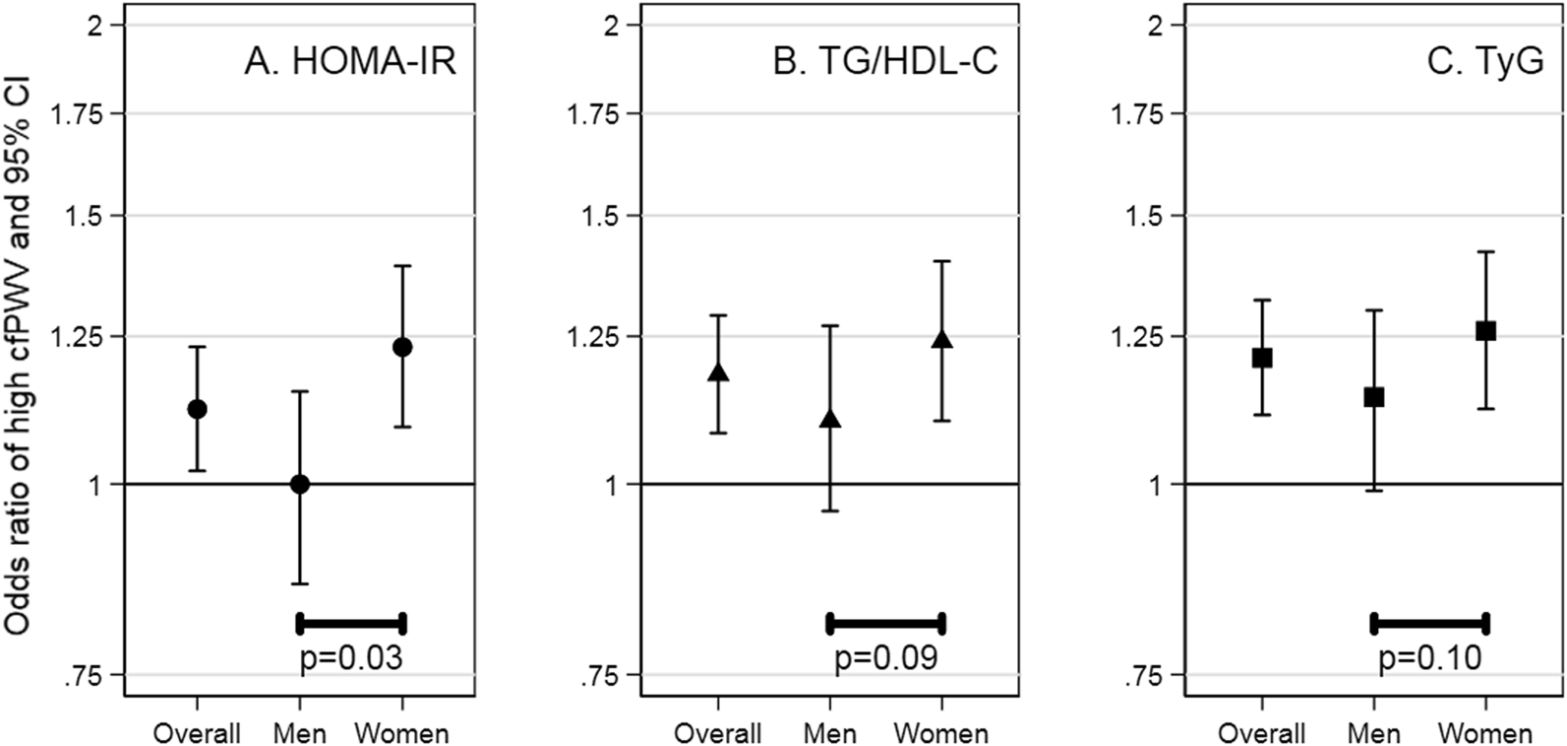
Cross-sectional association of insulin resistance indexes with high aortic stiffness in adults ages 67–90. *HOMA*-*IR* homeostatic model assessment of insulin resistance, *TG/HDL*-*C* triglyceride to high-density lipoprotein cholesterol ratio, *TyG* triglyceride and glucose index, *cfPWV* carotid-femoral pulse wave velocity. The odds ratio and 95% CI are interpreted as the odds of high (> 75th percentile), vs. non-high, aortic stiffness per standard deviation increment in insulin resistance index. The test for interaction is the p-value for the product term of insulin resistance index, that has been standardized, and gender. Estimates are adjusted for age, gender (except for gender-specific estimates), and race/study-site. The standard deviation was 1.6 for HOMA-IR, 1.3 for TG/HDL-C, and 0.4 for TyG

In the cross-sectional analysis, higher HOMA-IR, higher TG/HDL-C, and higher TyG were associated with higher odds of high aortic stiffness. Specifically, the odds of high aortic stiffness was higher per standard unit of HOMA-IR (OR: 1.12 (95% CI 1.02, 1.23)), TG/HDL-C (OR: 1.18 (95% CI 1.08, 1.29)), and TyG (OR: 1.21 (95% CI 1.11, 1.32)). The estimates were all higher for women than men for HOMA-IR, TG/HDL-C, and TyG, but the differences were not all statistically significant (p = 0.03, p = 0.09, and p = 0.10, respectively) (Fig. 2; Additional file 1: TableS5).

**Fig. 3:**
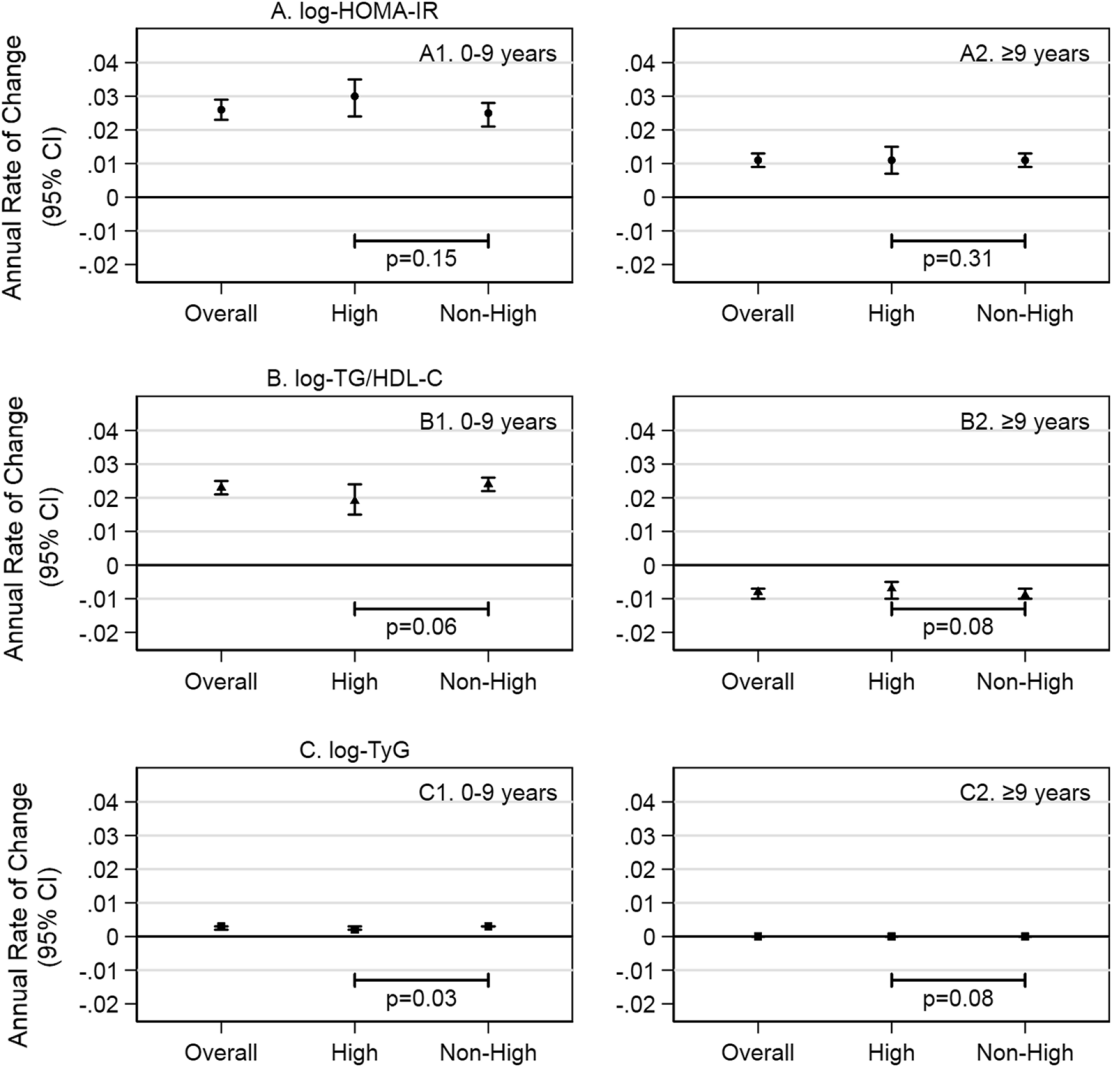
Association of high aortic stiffness and change in insulin resistance indexes in adults ages 67–90. *HOMA*-*IR* homeostatic model assessment of insulin resistance, *TG/HDL*-*C* triglyceride to high-density lipoprotein cholesterol ratio, *TyG* triglyceride and glucose index, *P75* 75th percentile. The annual rate of change and 95% CI are interpreted as the change in log-transformed insulin resistance index per year. The test for interaction is the p-value of the product term of time in study and high (> 75th percentile) aortic stiffness. Estimates are adjusted for age, gender, and race/study-site. Time in study was the time from Visit 1 to Visit 4 or Visit 5

In the prospective analysis, the annual rates of change were dissimilar for log-HOMA-IR, log-TG/HDL-C, and log-TyG. For log-HOMA-IR, percent change per year from baseline to 9 years was an increase of 2.6% (95% CI 2.3%, 2.9%)) followed by an increase of 1.1% (95% CI 0.9%, 1.3%)) from 9 years onward. For log-TG/HDL-C, percent change per year from baseline to 9 years was 2.3% (95% CI 2.1%, 2.5%)) but − 0.8% (95% CI − 1.0%, − 0.7%) from 9 years onward. For log-TyG there was a minimal increase from baseline to 9 years (percent change per year: 0.3% (95% CI 0.2%, 0.3%)) followed by no appreciable change from about 9 years onward (percent change per year: 0% (95% CI 0%, 0%)) (Fig. 3; Additional file 1: Table S6).

In the prospective analysis, there were differences between participants with high, compared to non-high, aortic stiffness, but the differences did not indicate a faster rate of change in the log-transformed index. For log-HOMA-IR, participants with high, compared to non-high, aortic stiffness had a faster rate of change from baseline to 9 years (high vs. non-high: 3.0% (95% CI 2.4%, 3.5%) vs. 2.5% (95% CI 2.1%, 2.8%); p = 0.15) and a similar rate of change from about 9 years onward (high vs. non-high: 1.1% (95% CI 0.7%, 1.5%) vs. 1.1% (95% CI 0.9%, 1.3%); p = 0.31). For log-TG/HDL-C, participants with high, compared to non-high, aortic stiffness had a positive rate of change from baseline to 9 years (high vs. non-high: 1.9% (1.5%, 2.4%) vs. 2.4% (2.2%, 2.6%); p = 0.06) then a negative rate of change from about 9 years onward (high vs. non-high: − 0.7% (− 1.0%, − 0.5%) vs. − 0.9% (− 1.0%, − 0.7%); p = 0.08). For log-TyG, participants with high and non-high aortic stiffness had a minimal rate of change from baseline to 9 years (high vs. non-high: 0.2% (0.2%, 0.3%) vs. 0.3% (0.3%, 0.3%); p = 0.03) and similarly no change from about 9 years onward (high vs. non-high: 0% (0%, 0%) vs. 0% (0%, 0%); p = 0.08) (Fig. 3; Additional file 1: Table S6).

We examined the cross-sectional analysis by percent change in abdominal adiposity from Visit 4 to Visit 5; we hypothesized that abdominal adiposity accumulated by late-life modified the effect of insulin resistance on aortic stiffness. Similar to the main analysis, aortic stiffness was higher per standard unit of HOMA-IR, TG/HDL-C, and TyG. The estimates of association were observed to be higher in participants who gained, as opposed to lost, waist girth, for HOMA-IR, TG/HDL-C, and TyG, but the confidence intervals overlapped, suggesting the differences in the estimates of association were not statistically significant (Additional file 1: Table S7).

We hypothesized that abdominal adiposity accumulated during mid-life modified the effect of insulin resistance, since mid-life, on aortic stiffness and thus examined the rate of temporal change in the log-transformed indexes by percent change in abdominal adiposity from examination Visit 3 to Visit 4. Similar to the overall analysis, the annual rates of change were dissimilar for log-HOMA-IR, log-TG/HDL-C, and log-TyG, but contrary to our expectation the annual rates of change by abdominal adiposity were not differential with respect to high, compared to non-high aortic stiffness, for log-HOMA-IR, log-TG/HDL-C, or log-TyG. Participants who gained waist girth and lost waist girth had similar estimates of association (Additional file 1: Table S8).

## Discussion

Cross-sectionally, higher HOMA-IR, higher TG/HDL-C, and higher TyG were associated with aortic stiffness in older adults without diabetes. However, higher aortic stiffness in older adults was not associated with faster annual rates of change in log-HOMA-IR, log-TG/HDL-C, or log-TyG from mid-life.

### Cross-sectional association: insulin resistance index and aortic stiffness

Prior studies have reported a cross-sectional relationship between insulin resistance and arterial stiffness, indicating that higher insulin resistance is associated with higher arterial stiffness, using similar index measures [8–11, 25–32] and similar arterial stiffness measures [8–11, 26, 27, 31]. However, exclusions for diabetes were not always clear or always included; and indexes reflected peripheral insulin resistance or hepatic insulin resistance but not always both. We therefore confirmed a relationship between insulin resistance and aortic stiffness in older adults without diabetes, using indexes that reflect more than one aspect of insulin resistance.

There has been a prior study that reported a cross-sectional relationship between insulin resistance and aortic stiffness (> 75th percentile) by gender [29]. This study found the effect size was stronger in men than women in middle adulthood, whereas in contrast our study found the effect size was, although not robust, stronger in women than men in older adulthood. Women have less visceral adipose tissue before menopause, but more visceral adipose tissue after menopause with the decline of endogenous estrogens [33, 34]. Men tend to have more visceral adipose tissue than women [33], but whether there is a shift in visceral adipose tissue is less clear. Differences in body composition may lead to differences in insulin resistance, subsequently leading to a difference in the effect of aortic stiffness seen in women and men.

### Prospective association: insulin resistance index, since mid-life, and aortic stiffness

We did not observe a steeper rate of change in log-HOMA-IR, log-TG/HDL-C, or log-TyG since mid-life with respect to aortic stiffness. Change in insulin resistance may be minimal in the absence of weight change [35]; or obscured by the lack of standardization of insulin assays that hinder the comparison of assays over time [15]. However, a recent study has reported an association between an increase in long-term glucometabolic impairment and an increase in aortic stiffness associated with hemoglobin A1c and HOMA-IR [36]. Recent reports identified associations of aortic stiffness with dysregulation in various metabolic pathways in the setting of type 2 diabetes. Treatment with an incretin mimetic was observed to improve carotid-femoral PWV in individuals with newly diagnosed type 2 diabetes [37] and serum levels of the acute phase reactant lipopolysaccharide-binding protein were shown to be associated with aortic PWV in patients with type 2 diabetes, especially in men [38]. Administration of sodium glucose co-transporter 2 inhibitor reduced aortic stiffness in type 2 diabetic female mice (db/db) [39]. Thus, although insulin resistance may contribute to aortic stiffness, we did not have support based on our study.

Given that accumulated abdominal adiposity is complex to capture, we observed that there may be a difference in estimates of association between participants who gain, as opposed to lose, waist girth, but this difference was not statistically significant cross-sectionally or prospectively. We add to our understanding of the role of abdominal adiposity on the relationship between insulin resistance and aortic stiffness by assessing abdominal adiposity at more than one time point.

### Implications for use of insulin resistance indexes in older adults

We found non-linearity in the temporal patterns of log-HOMA-IR, log-TG/HDL-C, and log-TyG. For log-HOMA-IR, there was an increase during middle adulthood (from baseline over the ensuing 9 years), followed by a slower increase into older adulthood. For log-TG/HDL-C, there was an increase during middle adulthood (from baseline over the ensuing 9 years), followed by a decrease in older adulthood. For log-TyG, there was a minimal increase during middle adulthood (from baseline over the following 9 years), then no change in older adulthood. The patterns seen in TG/HDL-C and TyG are consistent with the patterns seen in their constituent analytes; for example, HDL-C has been shown to increase with age and triglyceride has been shown to decrease with age, respectively, possibly reflecting factors such as weight loss, physical activity patterns, smoking cessation, and habitual alcohol consumption [40–44]. This discrepancy invites questions about the use of insulin resistance indexes in older adults. Our observations suggest that TG/HDL-C and TyG may be influenced by pathways different from those of HOMA-IR among older adults without diabetes.

### Limitations

Our results should be considered in the context of several limitations. Temporality cannot be established in a cross-sectional study. However, we assumed the temporality of the exposure and the outcome supported by an understanding of the role of hyperinsulinemia and hyperglycemia, that contribute to the formation of advanced glycation end-products that reduce arterial elasticity through arterial remodeling [45, 46] Informative censoring due to diabetes and death are potential sources of bias in a prospective study such as ours. However, we estimated the potential bias from informative censoring due to diabetes and death using shared parameter models and deemed the associated bias to be minimal, suggesting that the observed results may underestimate the annual rate of change in insulin resistance indexes.

## Conclusions

Among older adults without diabetes, higher HOMA-IR, higher TG/HDL-C, and higher TyG were associated with higher aortic stiffness, consistent with an association between insulin resistance and aortic wall remodeling and stiffening. However, high aortic stiffness was not preceded by a faster annual rate of change in log-HOMA-IR, log-TG/HDL-C, or log-TyG since mid-life. The hypothesized role of insulin resistance in aortic wall remodeling over the life course requires further study.

## Supplementary information

**Additional file 1.** This file contains the supplemental tables and supplemental methods that are referenced in the study.

## Data Availability

The data to support the findings of this study are available from the Atherosclerosis Risk in Communities Study. The data are available upon request from the authors and permission from the Atherosclerosis Risk in Communities Study.

## Abbreviations

HOMA-IR: homeostatic model assessment of insulin resistance
TG/HDL-C: triglyceride to high-density lipoprotein cholesterol ratio
TyG: triglyceride and glucose index
cfPWV: carotid-femoral pulse wave velocity
