# Supplementary material for: Association of insulin resistance, from mid-life to late-life, with aortic stiffness in late-life: the Atherosclerosis Risk in Communities Study
Source: Cardiovasc Diabetol. 2020 Jan 28;19:11. doi: 10.1186/s12933-020-0986-y (PMC6986071; doi:10.1186/s12933-020-0986-y)
Supplement: Supplementary file 1 — Additional file 1. This file contains the supplemental tables and supplemental methods that are referenced in the study. [file 12933_2020_986_MOESM1_ESM.docx]

Table S1. Assays by visit

| Visit | Year | Specimen | Lab | Instrument | Method |
| --- | --- | --- | --- | --- | --- |
| Glucose* | |  |  |  |  |
| Visit 1 | 1987-89 | Serum | UMN | Coulter Dacos | Coulter DACOS using hexokinase/ glucose-6-phosphate dehydrogenase enzyme-based reagent |
| Visit 2 | 1990-92 | Serum | UMN | Coulter Dacos | Coulter DACOS using hexokinase/ glucose-6-phosphate dehydrogenase enzyme-based reagent |
| Visit 3 | 1993-95 | Plasma | BCM | Roche Cobas Fara II | Enzymatic (hexokinase/ glucose-6-phosphate dehydrogenase) |
| Visit 4 | 1996-98 | Plasma | BCM | Roche Hitachi 911 | Enzymatic (hexokinase/ glucose-6-phosphate dehydrogenase) |
| Visit 5 | 2011-13 | Plasma | BCM | Beckman Olympus AU 480 | Enzymatic |
| Insulin |  |  |  |  |  |
| Visit 1 | 1987-89 | Serum | UMN | 125-Insulin kit; Cambridge Medical Diagnosis | Radioimmunoassay |
| Visit 2 | 1990-92 | ----- | ----- | ----- | ----- |
| Visit 3 | 1993-95 | ----- | ----- | ----- | ----- |
| Visit 4 | 1996-98 | Serum or plasma | BCM | Enzymun-Test Insulin; Boehringer Mannheim | Enzyme-linked immunosorbent assay |
| Visit 5 | 2011-13 | Serum or plasma | BCM | Elecsys Insulin kit; Roche | Electrochemiluminescence immunoassay |
| Triglyceride* | |  |  |  |  |
| Visit 1 | 1987-89 | Plasma | BCM | Roche Cobas Bio | Enzymatic |
| Visit 2 | 1990-92 | Plasma | BCM | Roche Cobas Bio | Enzymatic |
| Visit 3 | 1993-95 | Plasma | BCM | Roche Cobas Fara II | Enzymatic |
| Visit 4 | 1996-98 | Plasma | BCM | Roche Hitachi 911 | Enzymatic |
| Visit 5 | 2011-13 | Plasma | BCM | Beckman Olympus AU 480 | Enzymatic |
| High density lipoprotein cholesterol* | | | |  |  |
| Visit 1 | 1987-89 | Plasma | BCM | Roche Cobas Bio | Precipitation |
| Visit 2 | 1990-92 | Plasma | BCM | Roche Cobas Bio | Precipitation |
| Visit 3 | 1993-95 | Plasma | BCM | Roche Cobas Fara II | Precipitation |
| Visit 4 | 1996-98 | Plasma | BCM | Roche Hitachi 911 | Precipitation |
| Visit 5 | 2011-13 | Plasma | BCM | Beckman Olympus AU 480 | Direct |

**Abbreviations:** UMN, University of Minnesota; BCM, Baylor College of Medicine. **Footnote:** *Adapted from a prior report by Parrinello et al: Clin Chem. 2015 Jul; 61(7): 938-947.

Table S2. Characteristics of men and women ages 67-90 (n=2,571) by quartiles of TG/HDL-C (Visit 5, 2011-2013).

|  | TG/HDL-C | | | | Overall  Mean ± SE or n(%) |
| --- | --- | --- | --- | --- | --- |
|  | Quartile 1  Mean ± SE or n(%) | Quartile 2  Mean ± SE or n(%) | Quartile 3  Mean ± SE or n(%) | Quartile 4  Mean ± SE or n(%) |  |
| Demographic |  |  |  |  |  |
| Age, years | 75 ± 0.2 | 75 ± 0.2 | 76 ± 0.2 | 75 ± 0.2 | 75 ± 0.1 |
| Men | 189 (30) | 229 (35) | 229 (36) | 310 (48) | 957 (37) |
| African American | 145 (23) | 118 (18) | 107 (17) | 63 (10) | 433 (17) |
| Anthropometric |  |  |  |  |  |
| Waist circumference, cm | 92 ± 0.4 | 95 ± 0.4 | 98 ± 0.4 | 102 ± 0.4 | 97 ± 0.2 |
| BMI, kg/m^2^ | 25 ± 0.2 | 26 ± 0.2 | 27 ± 0.2 | 29 ± 0.2 | 27 ± 0.1 |
| Hemodynamic |  |  |  |  |  |
| SBP, mmHg | 130 ± 0.7 | 130 ± 0.7 | 130 ± 0.7 | 131 ± 0.7 | 130 ± 0.3 |
| DBP, mmHg | 66 ± 0.4 | 66 ± 0.4 | 66 ± 0.4 | 67 ± 0.4 | 66 ± 0.2 |
| MAP, mmHg | 87 ± 0.5 | 87 ± 0.4 | 88 ± 0.4 | 88 ± 0.4 | 88 ± 0.2 |
| Heart rate, beats per minute | 64 ± 0.4 | 64 ± 0.4 | 64 ± 0.4 | 64 ± 0.4 | 64 ± 0.2 |
| Blood pressure medication | 378 (60) | 398 (61) | 424 (66) | 440 (68) | 1640 (64) |
| Behavioral |  |  |  |  |  |
| Current smoker | 31 (5) | 40 (6) | 35 (6) | 35 (5) | 141 (6) |
| Current drinker | 375 (60) | 371 (58) | 330 (52) | 351 (55) | 1427 (56) |
| Former smoker | 283 (49) | 288 (48) | 290 (49) | 314 (52) | 1175 (49) |
| Former drinker | 134 (22) | 151 (24) | 154 (24) | 176 (28) | 615 (24) |
| cfPWV, cm/s |  |  |  |  |  |
| Men | 1133 ± 22 | 1104 ± 19 | 1168 ± 20 | 1160 ± 17 | 1143 ± 9 |
| Women | 1084 ± 14 | 1099 ± 14 | 1120 ± 14 | 1158 ± 15 | 1112 ± 7 |
| Overall | 1099 ± 12 | 1101 ± 11 | 1137 ± 11 | 1159 ± 11 | 1124 ± 6 |
| High cfPWV, >75th percentile |  |  |  |  |  |
| Men | 58 (31) | 47 (21) | 63 (28) | 85 (27) | 253 (26) |
| Women | 96 (22) | 93 (22) | 96 (23) | 102 (30) | 387 (24) |
| Overall | 154 (24) | 140 (22) | 159 (25) | 187 (29) | 640 (25) |

**Abbreviations:** BMI, body mass index; SBP, systolic blood pressure; DBP, diastolic blood pressure; MAP, mean arterial pressure; cfPWV, carotid-femoral pulse wave velocity; TG/HDL-C, triglyceride to high-density lipoprotein cholesterol ratio. **Footnotes:** Characteristics are defined by either the mean and standard error or the frequency and percent. For TG/HDL-C quartiles, the lower and upper limits are: [0.03, 1.36) for quartile 1; [1.36, 1.95) for quartile 2; [1.95, 2.84) for quartile 3; and [2.84, 9.34] for quartile 4.Table S3. Characteristics of men and women ages 67-90 (n=2,571) by quartiles of TyG (Visit 5, 2011-2013).

|  | TyG | | | | Overall  Mean ± SE  or n(%) |
| --- | --- | --- | --- | --- | --- |
|  | Quartile 1  Mean ± SE  or n(%) | Quartile 2  Mean ± SE  or n(%) | Quartile 3  Mean ± SE  or n(%) | Quartile 4  Mean ± SE  or n(%) |  |
| Demographic |  |  |  |  |  |
| Age, years | 75 ± 0.2 | 75 ± 0.2 | 76 ± 0.2 | 75 ± 0.2 | 75 ± 0.1 |
| Men | 283 (44) | 228 (35) | 201 (31) | 245 (38) | 957 (37) |
| African American | 155 (24) | 114 (18) | 100 (15) | 64 (10) | 433 (17) |
| Anthropometric |  |  |  |  |  |
| Waist circumference, cm | 94 ± 0.5 | 94 ± 0.4 | 98 ± 0.4 | 101 ± 0.4 | 97 ± 0.2 |
| BMI, kg/m^2^ | 26 ± 0.2 | 26 ± 0.2 | 27 ± 0.2 | 28 ± 0.2 | 27 ± 0.1 |
| Hemodynamic |  |  |  |  |  |
| SBP, mmHg | 129 ± 0.7 | 129 ± 0.7 | 130 ± 0.7 | 132 ± 0.7 | 130 ± 0.3 |
| DBP, mmHg | 66 ± 0.4 | 66 ± 0.4 | 67 ± 0.4 | 67 ± 0.4 | 67 ± 0.2 |
| MAP, mmHg | 87 ± 0.5 | 87 ± 0.4 | 88 ± 0.5 | 89 ± 0.4 | 88 ± 0.2 |
| Heart rate, beats per minute | 63 ± 0.4 | 63 ± 0.4 | 65 ± 0.4 | 65 ± 0.4 | 64 ± 0.2 |
| Blood pressure medication | 394 (62) | 385 (60) | 413 (64) | 448 (70) | 1640 (64) |
| Behavioral |  |  |  |  |  |
| Current smoker | 37 (6) | 40 (6) | 31 (5) | 33 (5) | 141 (6) |
| Current drinker | 342 (55) | 374 (58) | 340 (53) | 371 (59) | 1427 (56) |
| Former smoker | 296 (51) | 281 (47) | 293 (48) | 305 (52) | 1175 (49) |
| Former drinker | 162 (26) | 140 (22) | 162 (25) | 151 (24) | 615 (24) |
| cfPWV, cm/s |  |  |  |  |  |
| Men | 1131 ± 19 | 1112 ± 18 | 1161 ± 21 | 1171 ± 19 | 1143 ± 9 |
| Women | 1101 ± 16 | 1077 ± 14 | 1118 ± 14 | 1154 ± 14 | 1112 ± 7 |
| Overall | 1115 ± 12 | 1089 ± 11 | 1131 ± 11 | 1161 ± 11 | 1124 ± 6 |
| High cfPWV, >75th percentile |  |  |  |  |  |
| Men | 83 (29) | 51 (22) | 50 (25) | 69 (28) | 253 (26) |
| Women | 80 (23) | 83 (20) | 106 (24) | 118 (30) | 387 (24) |
| Overall | 163 (26) | 134 (21) | 156 (24) | 187 (29) | 640 (25) |

**Abbreviations:** BMI, body mass index; SBP, systolic blood pressure; DBP, diastolic blood pressure; MAP, mean arterial pressure; cfPWV, carotid-femoral pulse wave velocity; TyG, triglyceride and glucose index. **Footnotes:** Characteristics are defined by either the mean and standard error or the frequency and percent. For TyG, the lower and upper limits are: [6.92, 8.30) for quartile 1; [8.30, 8.57) for quartile 2; [8.57, 8.88) for quartile 3; and [8.88, 10.03] for quartile 4.

Table S4. Cross-sectional association of insulin resistance indexes with aortic stiffness in men and women ages 67-90 (Visit 5, 2011-2013).

|  |  | HOMA-IR | TG/HDL-C | TyG |
| --- | --- | --- | --- | --- |
|  | n | Difference (95% CI) in cfPWV (cm/s) per SD | Difference (95% CI) in cfPWV (cm/s) per SD | Difference (95% CI) in cfPWV (cm/s) per SD |
| Overall | 2571 | 16 (6,27) | 29 (18,40) | 32 (22,42) |
| Men | 957 | 8 (-9,25) | 24 (8,40) | 32 (16,48) |
| Women | 1614 | 23 (10,37) | 32 (18,46) | 30 (17,43) |
| Test for interaction | 2571 | p=0.23 | p=0.36 | p=0.94 |

**Abbreviations:** HOMA-IR, homeostatic model assessment of insulin resistance; TG/HDL-C, triglyceride to high-density lipoprotein cholesterol ratio; TyG, triglyceride and glucose index; cfPWV, carotid-femoral pulse wave velocity. **Footnotes:** The difference and 95% CI are interpreted as the difference in aortic stiffness per standard deviation (per SD) increment in insulin resistance index. The test for interaction is the p-value of the product term of insulin resistance index, that has been standardized, and gender. Estimates are adjusted for age, gender (except for gender-specific estimates), and race/study site. The standard deviation was 1.6 for HOMA-IR, 1.3 for TG/HDL-C, and 0.4 for TyG.

Table S5. Cross-sectional association of insulin resistance indexes with high (>75^th^-percentile) aortic stiffness in men and women ages 67-90 (Visit 5, 2011-2013).

|  |  | HOMA-IR | TG/HDL-C | TyG |
| --- | --- | --- | --- | --- |
|  | n | Odds Ratio (95% CI) for high cfPWV (>75^th^ percentile) per SD | Odds Ratio (95% CI) for high cfPWV (>75^th^ percentile) per SD | Odds Ratio (95% CI) for high cfPWV (>75^th^ percentile) per SD |
| Overall | 2571 | 1.12 (1.02,1.23) | 1.18 (1.08,1.29) | 1.21 (1.11,1.32) |
| Men | 957 | 1.00 (0.86,1.15) | 1.10 (0.96,1.27) | 1.14 (0.99,1.3) |
| Women | 1614 | 1.23 (1.09,1.39) | 1.24 (1.10,1.40) | 1.26 (1.12,1.42) |
| Test for interaction | 2571 | p=0.03 | p=0.09 | p=0.10 |

**Abbreviations:** HOMA-IR, homeostatic model assessment of insulin resistance; TG/HDL-C, triglyceride to high-density lipoprotein cholesterol ratio; TyG, triglyceride and glucose index; cfPWV, carotid-femoral pulse wave velocity. **Footnotes:** The odds ratio and 95% CI are interpreted as the odds of high, vs non-high, aortic stiffness per standard deviation (per SD) increment in insulin resistance index. The test for interaction is the p-value of the product term of insulin resistance index, that has been standardized, and gender. Estimates are adjusted for age, gender (except for gender-specific estimates), and race/study-site. The standard deviation was 1.6 for HOMA-IR, 1.3 for TG/HDL-C, and 0.4 for TyG.

Table S6. Association of aortic stiffness (Visit 5, 2011-2013) and change in insulin resistance indexes (Visit 1, 1987-1989 to Visit 5, 2011-2013) in men and women ages 67-90 (Visit 5, 2011-2013).

|  |  | log-HOMA-IR | |  | log-TG/HDL-C | |  | log-TyG | |
| --- | --- | --- | --- | --- | --- | --- | --- | --- | --- |
|  |  | Time in study  0 to 9 years | Time in study  ≥9 years |  | Time in study  0 to 9 years | Time in study  ≥9 years |  | Time in study  0 to 9 years | Time in study  ≥9 years |
|  | n | Annual rate of  change (95% CI) | Annual rate of  change (95% CI) |  | Annual rate of  change (95% CI) | Annual rate of  change (95% CI) |  | Annual rate of  change (95% CI) | Annual rate of  change (95% CI) |
| Overall | 2350 | 0.026  (0.023,0.029) | 0.011  (0.009,0.013) |  | 0.023  (0.021,0.025) | -0.008  (-0.010,-0.007) |  | 0.003  (0.002,0.003) | 0  (0,0) |
| High cfPWV ($>$P75) | 587 | 0.030  (0.024,0.035) | 0.011  (0.007,0.015) |  | 0.019  (0.015,0.024) | -0.007  (-0.010,-0.005) |  | 0.002  (0.002,0.003) | 0  (0,0) |
| Non-High cfPWV ($\leq$P75) | 1763 | 0.025  (0.021,0.028) | 0.011  (0.009,0.013) |  | 0.024  (0.022,0.026) | -0.009  (-0.010,-0.007) |  | 0.003  (0.003,0.003) | 0  (0,0) |
| Test for  interaction | 2350 | 0.15 | 0.31 |  | 0.06 | 0.08 |  | 0.03 | 0.08 |

**Abbreviations:** HOMA-IR, homeostatic model assessment of insulin resistance; TG/HDL-C, triglyceride to high-density lipoprotein cholesterol ratio; TyG, triglyceride and glucose index; P75, 75^th^ percentile. **Footnotes:** The annual rate of change and 95% CI are interpreted as the change in log-transformed insulin resistance index per year. The test for interaction is the p-value of the product term of time in study and high aortic stiffness. Estimates are adjusted for age, gender, and race/study-site. Time in study was the time from Visit 1 to Visit 4 or Visit 5.

Table S7. Cross-sectional association of insulin resistance indexes with aortic stiffness in men and women ages 67-90 (Visit 5, 2011-2013), by percent change in abdominal adiposity (from Visit 4,1996-1998 to Visit 5, 2011-2013).

|  |  | HOMA-IR | TG/HDL-C | TyG |
| --- | --- | --- | --- | --- |
|  | n | Difference (95% CI) in  cfPWV (cm/s) per SD | Difference (95% CI) in  cfPWV (cm/s) per SD | Difference (95% CI) in  cfPWV (cm/s) per SD |
| Percent Gain (Percent change $\geq$0%) | 1396 | 20 (6,33) | 35 (21,48) | 33 (20,46) |
| Percent Loss (Percent change $<$0%) | 1019 | 16 (-3,35) | 16 (-3,34) | 27 (10,44) |

**Abbreviations:** HOMA-IR, homeostatic model assessment of insulin resistance; TG/HDL-C, triglyceride to high-density lipoprotein cholesterol ratio; TyG, triglyceride and glucose index; cfPWV, carotid-femoral pulse wave velocity. **Footnotes:** The difference and 95% CI are interpreted as the difference in aortic stiffness per standard deviation (per SD) increment in insulin resistance index. Estimates are adjusted for age, gender (except for gender-specific estimates), and race/study site. The standard deviation was 1.6 for HOMA-IR, 1.3 for TG/HDL-C, and 0.4 for TyG. There are n=156 missing percent change in abdominal adiposity.

Table S8. Association of aortic stiffness (Visit 5, 2011-2013) and change in insulin resistance indexes (Visit 1, 1987-1989 to Visit 5, 2011-2013) in men and women ages 67-90 (Visit 5, 2011-2013), by percent change in abdominal adiposity (from Visit 3, 1993-1995 to Visit 4,1996-1998).

|  |  | log-HOMA-IR | |  | log-TG/HDL-C | |  | log-TyG | |
| --- | --- | --- | --- | --- | --- | --- | --- | --- | --- |
|  |  | Time in study  0 to 9 years | Time in study  $\geq$9 years |  | Time in study  0 to 9 years | Time in study  $\geq$9 years |  | Time in study  0 to 9 years | Time in study  $\geq$9 years |
|  | n | Annual rate of  change (95% CI) | Annual rate of  change (95% CI) |  | Annual rate of  change (95% CI) | Annual rate of  change (95% CI) |  | Annual rate of  change (95% CI) | Annual rate of  change (95% CI) |
| Percent Gain  ($\geq$0%) | 1635 | 0.029  (0.025,0.032) | 0.011  (0.009,0.013) |  | 0.025  (0.023,0.028) | -0.009  (-0.011,-0.008) |  | 0.003 (0.003,0.003) | 0  (0,0) |
| High cfPWV  ($>$P75) | 408 | 0.033  (0.026,0.039) | 0.01  (0.006,0.014) |  | 0.023  (0.017,0.028) | -0.009  (-0.012,-0.005) |  | 0.003 (0.002,0.003) | 0  (0,0) |
| Non-High cfPWV  ($\leq$P75) | 1227 | 0.027  (0.023,0.031) | 0.011  (0.009,0.014) |  | 0.026  (0.023,0.029) | -0.009  (-0.011,-0.007) |  | 0.003 (0.003,0.003) | 0  (0,0) |
| Test for  interaction | 1635 | 0.20 | 0.28 |  | 0.21 | 0.28 |  | 0.16 | 0.27 |
|  |  |  |  |  |  |  |  |  |  |
| Percent Loss  ($<$0%) | 669 | 0.02  (0.014,0.025) | 0.011  (0.008,0.015) |  | 0.017  (0.013,0.021) | -0.006  (-0.008,-0.003) |  | 0.002 (0.002,0.002) | 0  (0,0.001) |
| High cfPWV  ($>$P75) | 169 | 0.022  (0.011,0.034) | 0.013  (0.006,0.02) |  | 0.013  (0.005,0.021) | -0.004  (-0.009,0.001) |  | 0.002 (0.001,0.002) | 0  (0,0.001) |
| Non-High cfPWV  ($\leq$P75) | 500 | 0.019  (0.012,0.025) | 0.01  (0.006,0.015) |  | 0.019  (0.014,0.023) | -0.006  (-0.009,-0.004) |  | .002 (0.002,0.003) | 0  (0,0.001) |
| Test for interaction | 669 | 0.57 | 0.92 |  | 0.20 | 0.21 |  | 0.12 | 0.26 |

**Abbreviations:** HOMA-IR, homeostatic model assessment of insulin resistance; TG/HDL-C, triglyceride to high-density lipoprotein cholesterol ratio; TyG, triglyceride and glucose index; P75, 75^th^ percentile. **Footnotes:** The annual rate of change and 95% CI are interpreted as the change in log-transformed insulin resistance index per year. The test for interaction is the p-value of the product term of time in study and high aortic stiffness. Estimates are adjusted for age, gender, and race/study-site. Time in study was the time from Visit 1 to Visit 4 or Visit 5. There are n=46 missing percent change in waist circumference.

Methods S1. Exclusions for insulin resistance indexes at Visits 1, 4, and 5

Insulin resistance indexes were measured at Visits 1, 4, and 5. At Visit 1, we excluded: 1) prevalent diabetes or missing diabetes status at Visit 1 (n=1870 and n=148, respectively); 2) African American participants from the Minneapolis and Washington County field centers, Asian participants, and Native American participants due to small sample size (n=95); 3) age <45 years due to small sample size (n=41); and 4) missing covariates at Visit 1 (n=320). At Visits 1, 4, and 5, we excluded participants with: 1) diabetes or missing diabetes status; 2) fasting <8 hours; and 3) analytes ±3 standard deviations from the mean. Then, we excluded missing either insulin resistance index at Visit 1 (n=770). After exclusions, insulin resistance indexes were available for 12,548 participants (of 15,792 participants at Visit 1).

Methods S2. Addressing informative censoring from diabetes and death using shared parameter models

**Introduction:** Our goal was to describe insulin resistance indexes from mid-life to late-life. We hypothesized that insulin resistance indexes increased from mid-life to late-life.

**Study Population:** The Atherosclerosis Risk in Communities Study is an ongoing prospective study of 15,792 adults 45 to 64 years old at baseline, with standardized examinations at Visit 1 (1987-89), Visit 2 (1990-92), Visit 3 (1993-95), Visit 4 (1996-98), and Visit 5 (2011-13). At Visit 1, we excluded: 1) prevalent diabetes or missing diabetes status at Visit 1 (n=1870 and n=148, respectively); 2) African American adults from the Minneapolis and Washington County field centers, Asian adults, and Native American adults due to small sample size (n=95); 3) age <45 years due to small sample size (n=41); and 4) missing covariates at Visit 1 (n=320). At Visits 1, 4, and 5, we excluded adults with: 1) diabetes or missing diabetes status; 2) fasting <8 hours; and 3) analytes ±3 standard deviations from the mean. Then, we excluded missing either insulin resistance index at Visit 1 (n=770). The analytic set included 12,548 adults.

**Methods:** Insulin resistance indexes included: the homeostatic model assessment of insulin resistance, calculated as HOMA-IR = (fasting glucose in mg/dL) x (fasting insulin in μU/mL) / 405; and the triglyceride to high-density lipoprotein cholesterol ratio, calculated as TG/HDL-C = (triglyceride in mg/dL) / (high-density lipoprotein cholesterol in mg/dL). Mean and median were used to describe insulin resistance indexes by time in study. Linear mixed effects models were used to estimate the rate of change. Shared parameter models were used to address potential bias introduced by informative censoring due to diabetes and death. Covariates, initially chosen for their role as predictors of insulin resistance, were selected using the likelihood ratio test. Selected covariates included time in study as a linear spline term due to non-linearity at 9 years. Other covariates at Visit 1 included: gender, race, waist circumference, systolic blood pressure, blood pressure lowering medicaitons, current smoker status, family history of diabetes, and leisure time index. The insulin resistance indexes were log-transformed due to skew. Estimates were thus interpreted as the change in log-units per year and, for ease of interpretation, re-expressed as the percent change per year [(e^β^ – 1) x 100].

**Results:** Mean age was 54 years, 56% (n=7,003) were female, and 23% (n=2,830) were African American. Mean time in study was 0, 3, 6, 9, and 24 years. For HOMA-IR, the median increased from 2.07 to 2.32 from baseline to 9 years in study; the median increased to 2.44 from 9 years to 24 years in study. For TG/HDL-C, the median increased from 2.21 to 2.47 from baseline to 9 years in study; the median decreased to 2.00 from 9 years to 24 years in study. For log-transformed HOMA-IR, the change per year was 0.019; after 9 years in study, the change per year was 0.010. The corresponding percent change per year before and after 9 years in study was 1.9% and 1.0%, respectively. For log-transformed TG/HDL-C, the change per year was 0.017; after 9 years in study, the change per year was -0.008. The corresponding percent change per year before and after 9 years was 1.7% and -0.8%, respectively.

There was evidence of informative censoring, but the associated bias was minimal. For log-transformed HOMA-IR, the rate of change before and after 9 years of 0.019 and 0.010 increased, respectively, to 0.023 and 0.014 after addressing informative censoring. For log-transformed TG/HDL-C, the rate of change before and after 9 years of 0.017 and -0.008 increased, respectively, to 0.018 and -0.007 after addressing informative censoring. Addressing bias indicated steeper rates of change, but on average, the magnitude of bias was ignorable.

**Conclusions:** In adults without diabetes, insulin resistance indexes increase from mid-life to late-life, which may be consistent with (occur together with) the accumulation of risk factors from mid-life to late-life. Understanding the temporal patterns of insulin resistance indexes can inform strategies for primary prevention.
